# Spatial and Temporal Distribution of Mosquito Species (Culicidae) in a Ramsar Site, Fetzara Lake (Annaba, Algeria)

**DOI:** 10.3390/insects16101057

**Published:** 2025-10-16

**Authors:** Amna Rouibi, Abdelhakim Rouibi, Rassim Khelifa

**Affiliations:** 1 Department of Biology, Faculty of Sciences, Badji Mokhtar University, Annaba 23000, Algeria; 2 Laboratory of Biology, Water and Environment (LBEE), The 8 Mai 1945 University, Street P.O. Box 401, Guelma 24000, Algeria; rouibi.ah@gmail.com; 3 Biology Department, Concordia University, 7141 Sherbrooke St. W., Montreal, QC H4B 1R6, Canada

**Keywords:** Culicidae, Diptera, wetland, inventory, Fetzara Lake, Annaba, Algeria

## Abstract

Mosquito community composition can differ spatially and temporally within the same wetlands. Understanding this spatiotemporal variation is crucial, particularly in wetlands of conservation importance. Here, we examine the diversity and community composition of Culicidae (Diptera) across four sites within Fetzara Lake, a large Ramsar site in Northeast Algeria. For two years, we conducted monthly field surveys across four sites (Northeast, Northwest, Southeast, and Southwest) from April 2021 to March 2023. During these surveys, we used ovitraps to sample mosquitoes and assess species richness as well as alpha and beta diversity. We identified seven mosquito species (*Aedes aegypti*, *Ae. albopictus*, *Ae. geniculatus*, *An. labranchiae*, *Culex perexiguus*, *Cx. pipiens*, and *Cs. longiareolata*). There was a clear dominance of *Culex pipiens* (Usutu and West Nile virus vector), which accounted for 74.3% of all samples, whereas *Aedes aegypti* was the least abundant (<1%). Species richness varied between five and six across sites. The Shannon index and beta diversity revealed significant variation in species diversity across sites and seasons, likely driven by local differences in environmental conditions. This study emphasizes the importance of local variation in environmental conditions in shaping ecological communities in space and time.

## Simple Summary

The distribution of mosquitoes can vary in space and time, even within the same wetland. In this study, we monitored mosquito diversity in a protected wetland in Northeast Algeria (Fetzara Lake) to understand how mosquito communities vary across different sites within the wetland and seasons. We conducted monthly mosquito sampling for two years (April 2021–March 2023) from four sites within the lake. We identified seven mosquito species, including the *Culex pipiens* (common vector of West Nile virus) and *Aedes aegypti* (common vector of dengue and Zika). *Culex pipiens* dominated our samples (74.3%), while *Aedes aegypti* was rare. We found differences in the diversity between sites and across seasons. The spatial difference likely reflected local differences in environmental conditions (e.g., water quality, vegetation). Overall diversity was similar in both years. Our findings reveal that mosquito communities can markedly vary within the same wetland across space and seasons, highlighting the need for targeted mosquito control in areas where disease-carrying species are most common.

## 1. Introduction

Mosquitoes (Diptera: Culicidae) are a key component of freshwater ecosystems, playing key ecosystem functions in freshwater food webs [1,2]. Both larvae and adults are important prey for a variety of taxa (e.g., aquatic invertebrates, fish, and amphibians) [3], including those of conservation concern [4]. The monitoring of mosquitoes in wetlands is crucial to understand key ecological processes in wetland ecosystems, which help inform ecological management, conservation efforts, and the control of vector-borne diseases [5]. In large wetlands, where physical, chemical, and biological conditions can vary substantially across space, mosquito assemblages can exhibit fine-scale differences in species composition and diversity. Such spatial heterogeneity in diversity of mosquitoes at the wetland level has received little research attention, particularly in wetlands of conservation importance. Thus, revealing these patterns can provide insights into improving existing monitoring schemes of vector-borne diseases [6,7].

North African wetlands are unique ecological systems, characterized by harsh dry and warm conditions [8,9]. The extensive droughts and seasonal fluctuations in water availability influence habitat conditions and ecological communities. Despite the critical role of North African wetlands for biodiversity (e.g., breeding and foraging sites for numerous waterbirds and macroinvertebrates), the mosquito assemblages of many of these wetlands remain poorly studied [10]. Unlike in many European regions where mosquito research is more extensive in both lotic and lentic sites, studies on Culicidae in North Africa have predominantly focused on lotic environments such as rivers and streams [11,12,13,14]. This knowledge gap is particularly evident in Ramsar-listed wetlands, which host a substantial number of insectivorous waterbirds yet lack baseline data on mosquito diversity and community composition. Establishing such a baseline is critical for assessing future shifts in mosquito populations driven by climate change and anthropogenic disturbances, including habitat fragmentation and altered hydrological regimes [15,16,17]. Given the recent outbreaks of the West Nile virus in the Mediterranean [18], understanding how mosquito communities vary locally is key to enhancing monitoring efforts and informing outbreak response strategies.

Mosquito abundance and community composition in wetlands are shaped by both abiotic and biotic factors. Water parameters such as temperature, pH, dissolved oxygen, and salinity as well as aquatic vegetation influence egg laying behavior and larval survival, acting as environmental filters that shape mosquito community composition [19,20,21]. Such environmental factors also influence biotic interactions—such as competition, predation, and host availability—which in turn shape mosquito community structure directly or indirectly [22,23,24,25]. Given the spatial heterogeneity of large wetlands [26,27], mosquito assemblages can vary even at small scales. Understanding this spatial variability is crucial for guiding ecological research, conservation, and vector management in sensitive wetland areas

In this study, we investigated the diversity and species composition of mosquitoes in Fetzara Lake, a large Ramsar site in Northeast Algeria. We performed field surveys where we used traps in four sites (NE, NW, SE, and SW) from April 2021 to March 2023. We estimated the species richness, diversity, as well as their seasonal patterns. We hypothesize that there is variation in richness and diversity of Culicidae within the wetland due to the large size of the wetland and the marked habitat complexity across its perimeter. We also predict that both studied years will not show a difference in species composition due to the similarity in their climatic conditions.

## 2. Materials and Methods

**Figure 1:**
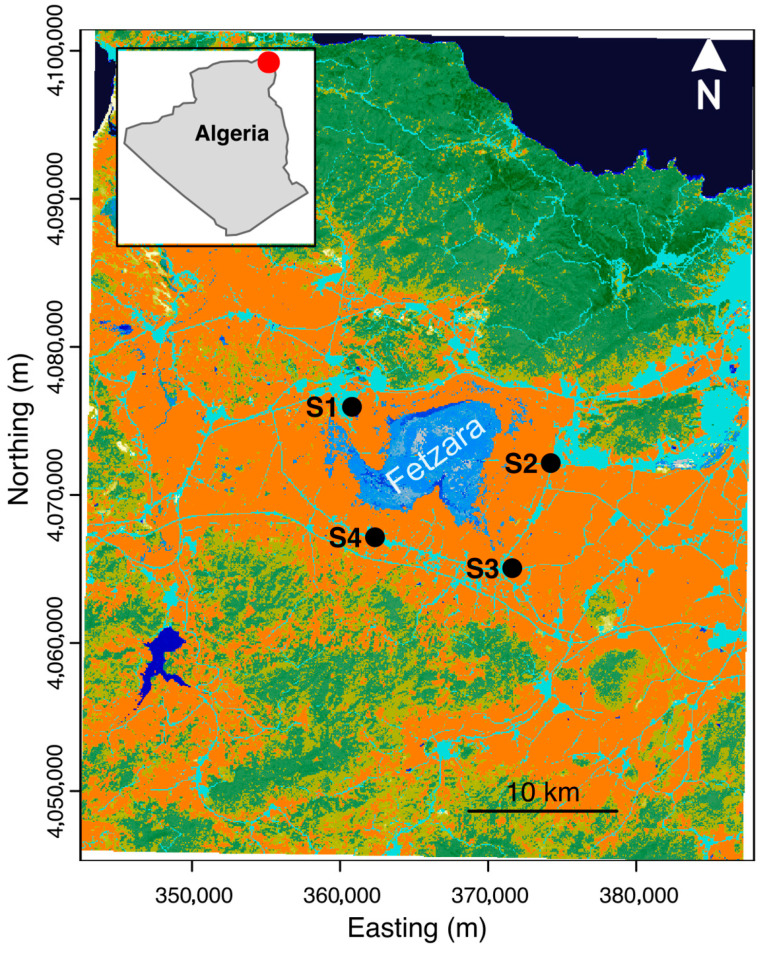
Map of our sampling sites in Fetzara Lake, Northeast Algeria. Northwest (NW: S1), Northeast (NE: S2), Southeast (SE: S3), and Southwest (SW: S4). The map was based on the Global land cover and land use 2020 (GLAD Landsat Analysis Ready Data).

Study area. Fetzara is a freshwater lake (altitude a.s.l.: 30 m), classified as a Ramsar site, located in Annaba region at the extreme northeast of Algeria (Figure 1), with a surface of 5800 hectares (58 km^2^) typically increasing by an additional 4000 ha (40 km^2^) as the land floods in the rainy winter season. The lake was surrounded by permanent and seasonal marshes and pools, and by irrigated and seasonally inundated agricultural land; a rare representative of natural wetlands in the Mediterranean region [28]. On the outskirts of the lake there are several towns: to the north, the commune of Berrahal, to the south the territories of the communes (municipalities) of El Eulma and Cheurfa and to the east Sidi Amar and Oued El Aneb [29]. This lake is fed by rainfall and rivers that also receive liquid effluents from peripheral urban and industrial areas. Its main rivers are Oued El Hout to the south, Oued Mellah to the west and Oued Zied to the northeast [30]. Its subhumid Mediterranean climate is characterized by two contrasting seasons, a cool, humid period of eight months (from October to May) and a hot, dry period of four months (from June to September) [31].

The site has ornithological importance as it hosts an average of 30,000 wintering waterbirds and serves as a breeding site for numerous species [30]. The site has thus potential relevance for zoonotic disease transmission, as birds can act as reservoirs for pathogens such as West Nile virus and Usutu virus [32], which may be vectored by mosquito species present in the area. The site is also characterized by intensive agricultural and pastoral activity [33]. In addition to these activities, the wastewater of the surrounding urban agglomerations constitutes an important source of pollution of the lake waters. The pollution concentrations vary spatially depending on the distance from the source of pollution and characteristics of the rainy and dry season [34,35]. We collected monthly temperature and precipitation data for the two sampling years (2021–2022 and 2022–2023) from a meteorological station in Annaba (latitude: 36.83329, longitude: 7.81849, altitude a.s.l.: 22 m), located 22 km east of Fetzara Lake (Appendix A).

Mosquito sampling. We collected samples using a method originally developed by Fay and Eliason [36]. Oviposition traps have proven effective for studying *Aedes* larvae and monitoring vector populations [37]. However, our previous research demonstrated that other mosquito species from other genera also prefer these traps [38]. The traps consisted of 10 L black plastic buckets [39]. In addition to species identification, we used ovitraps to assess species coexistence and conduct a [2] comparative analysis of species composition within the lake.

To collect larvae, we deployed 16 fenced ovitraps at four sites around the lake (NE, NW, SE, and SW; Figure 1), with each location containing four traps one meter apart. Sampling occurred twice a month over two years (April 2021–March 2023), resulting in a total of 768 samples. Traps remained stable and consistently contained water to ensure a suitable habitat for mosquito larvae. The lake’s favorable environmental conditions further enhanced its attractiveness for oviposition. Collected samples were placed in separate labeled containers, sorted by site and date, and transported to the laboratory for identification. After identification, samples were preserved in tubes containing 70% ethanol. Species identification was conducted using both the European mosquito identification key [40] and the African mosquito identification key [41].

Mosquito Diversity Analysis. Mosquito species count data were used to generate graphs illustrating species abundance and composition across different sites, months, and years. Diversity analyses were conducted using the vegan [42] package in R 4.3.3 [43]. Alpha diversity (mean species diversity) was quantified using the Shannon index via the ‘diversity()’ function. Beta diversity (variation in species composition across traps) was analyzed using non-metric Multidimensional Scaling (NMDS) with the metaMDS() function. The impact of site, year, and season on mosquito diversity was assessed using a Permutational Multivariate Analysis of Variance (PERMANOVA) with the adonis2() function of the vegan package. Here, seasons were defined according to the sampling months as follows: autumn (September–November), winter (December–February), spring (March–May), and summer (June–August). The Bray–Curtis dissimilarity matrix was calculated with the vegdist() function. The goodness of fit of the NMDS was assessed using a Shepard diagram generated with the goodness() function.

Statistical analysis. We used R 4.3.3 to conduct all statistical analyses [43]. We used Mann–Whitney U tests to check for differences in monthly air temperature, monthly precipitation, and Shannon index between years. We used Kruskal–Wallis tests to determine if Shannon index differed significantly between sites. We computed chi-square tests to assess whether the abundance of larvae differed significantly between species and sites. Values are mean ± SD.

## 3. Results

Richness. A total of seven species were recorded in the Fetzara Lake (*Aedes aegypti*, *Ae. albopictus*, *Ae. geniculatus*, *An. labranchiae*, *Cx. perexiguus*, *Cx. pipiens*, *Cs. longiareolata*), belonging to five genera. Richness varied between five and six across the four different sites. Four species were recorded in all sites, two species were recorded in two sites, whereas one species (*Aedes aegypti*) was recorded only in one site.

**Figure 2:**
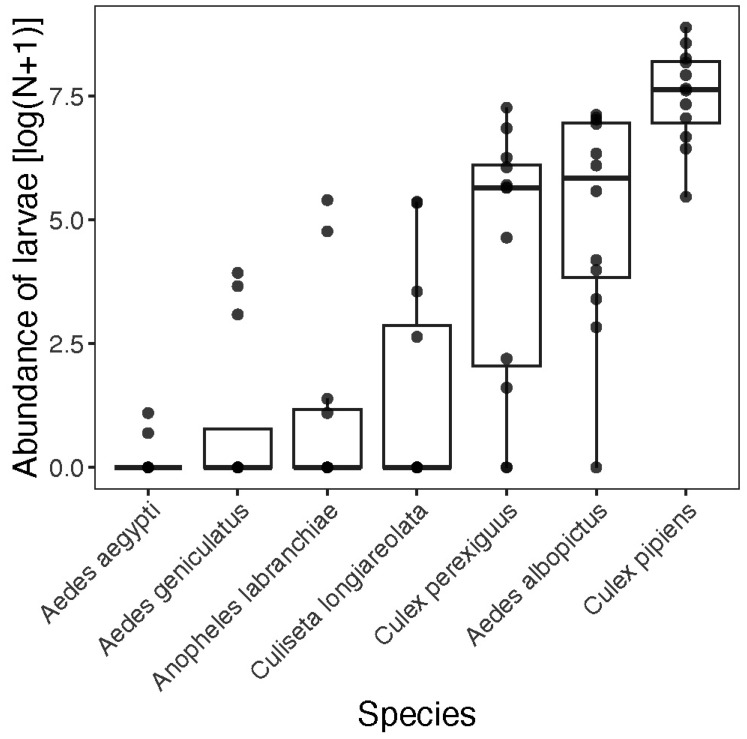
Abundance of mosquitoes trapped in Fetzara Lake, Northeast Algeria.

**Figure 3:**
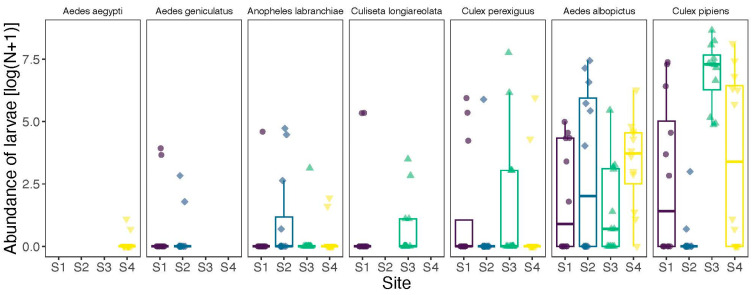
Abundance of mosquitoes trapped across four sites in the Fetzara Lake, Northeast Algeria. NW: Northwest (S1); NE: Northeast (S2); SE: Southeast (S3); and SW: Southwest (S4).

**Figure 4:**
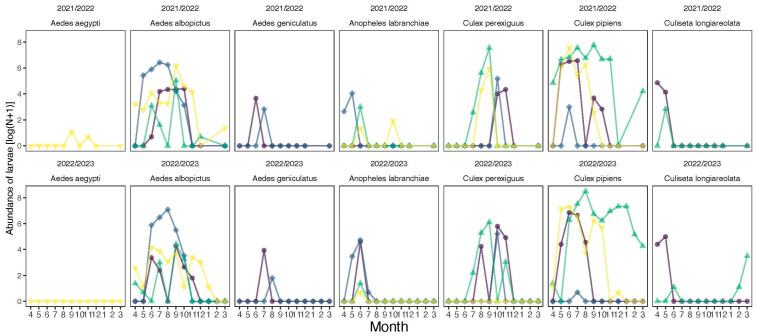
Seasonal pattern of abundance of mosquitoes trapped across four sites in the Fetzara Lake, Northeast Algeria in both years. Northwest (S1); NE: Northeast (S2); SE: Southeast (S3); and SW: Southwest (S4).

Abundance. Across the 16 ovitraps, we collected a total of 44,006 mosquitoes from seven different species in Fetzara Lake. A total of 20,112 (45.7%) were collected in 2021/2022, and 23,894 were collected in 2022/2023 (54.3%). There was a clear dominance of *Culex pipiens* in our samples, which accounted for 32,714 individuals (74%) (Figure 2), contributing to a significant difference in abundance among species (Chi-square test: χ^2^ = 135,072, df = 6, *p* < 0.0001). The distribution of mosquitoes varied significantly across sites (Chi-square test: χ^2^ = 23,662, df = 3, *p* < 0.0001) (Table 1; Figure 3). Mosquito abundance also varied significantly across months (Chi-square test: χ^2^ = 33,241, df = 11, *p* < 0.0001), highlighting temporal fluctuations in mosquito activity at Fetzara Lake (Figure 4). Four months (June–September) accounted for 72% of all individuals collected. The most abundant species (*Ae. albopictus*, *Cx. perexiguus*, and *Cx. pipiens*) were sampled across different seasons (except the winter) whereas other species were collected mostly during spring and summer (*Aedes aegypti*, *Ae. geniculatus*, *An. labranchiae*, and *Cs. longiareolata*).

Diversity. Shannon index was significantly different across sites (Kruskal–Wallis test: χ^2^ = 13.739, df = 3, *p* = 0.003). The highest Shannon index was recorded in site 3 (0.14 ± 0.25), followed by site 1 (0.11 ± 0.21), site 4 (0.10 ± 0.19), and site 2 (0.05 ± 0.15). The high standard deviations observed across all sites indicate substantial temporal variability in species diversity. Post hoc Dunn’s test revealed that Shannon index was significantly lower in site 2 compared to site 3 (*p* = 0.004) and site 4 (*p* = 0.01), but no significant differences were found among the other pairwise comparisons. In contrast, Shannon index did not differ significantly between years (Mann–Whitney U test: W = 12,644, *p* = 0.34).

**Figure 5:**
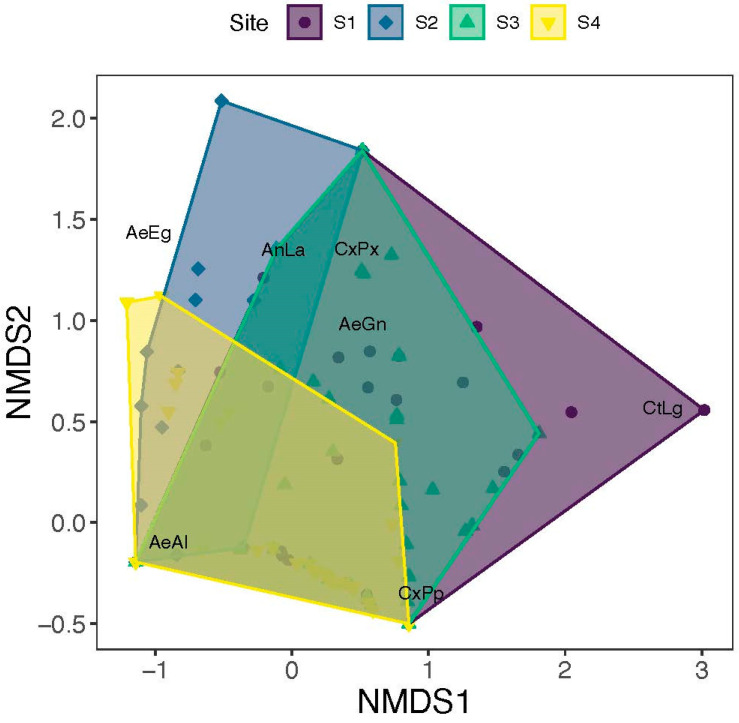
NMDS showing separation of samples by site. Northwest (S1); NE: Northeast (S2); SE: Southeast (S3); and SW: Southwest (S4).

**Figure 6:**
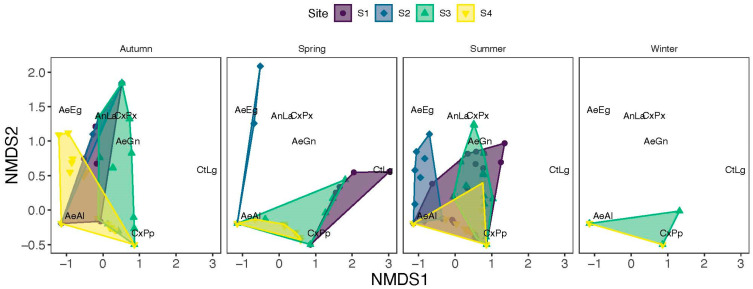
NMDS showing separation of samples by season and site. Autumn refers to September, October, and November; Winter refers to December, January, and February; Spring refers to March, April, and May; and summer refers to June, July, and August. Northwest (S1); NE: Northeast (S2); SE: Southeast (S3); and SW: Southwest (S4).

NMDS analysis showed a separation of samples due to sites (Figure 5; k = 2, stress = 0.08). PERMANOVA confirmed a significant impact of sites (R^2^ = 0.17, F_3,352_ = 27.3, *p* = 0.001) and year (R^2^ = 0.01, F_1,352_ = 3.60, *p* = 0.004) on the diversity of species. There was a marked overlap in the diversity of mosquitoes between site 1 and site 3 as well as site 4 and site 3, while Site 2 showed a weaker overlap with the other sites (Figure 5). Our NMDS analysis also showed a seasonal separation of samples (Figure 6), as revealed by a significant effect of season (R^2^ = 0.05, F_3,352_ = 8.20, *p* = 0.001). The significant interaction between sites and season shows that the overlap in mosquito diversity between sites differed between seasons (R^2^ = 0.06, F_7,352_ = 4.53, *p* = 0.001) (Figure 6).

## 4. Discussion

We investigated whether there was a spatial and seasonal variation in mosquito assemblages across different sites of a large wetland of conservation importance in North Africa. Seven mosquito species were identified in the wetland, with one species (*Culex pipiens*) dominating the assemblage. Our analysis revealed that the sampling location and season accounted for a significant portion of the variation in mosquito diversity, but no differences were observed across years. Of the seven recorded species of Culicidae at Fetzara Lake, five (*Aedes aegypti*, *Aedes albopictus*, *Anopheles labranchiae*, *Culex perexiguus*, and *Culex pipiens*) are particularly notable for their roles in disease transmission in North Africa. For instance, *Cx. pipiens* is known to be a vector of the Usutu and West Nile viruses [10]. This species composition corroborates the growing knowledge of Culicidae fauna in the Northeast Algeria in particular and the country in general.

The recorded richness represents 13.5% of the known Algerian diversity [44], and 8.75% of the North African species [10]. The species recorded in this study are relatively common in North Africa [10], and have been reported in previous studies [45,46,47]. The study of Culicidae in Algeria started with the research of Sergent and Sergent [48], who documented the presence of nine species. Over the 20th century, additional species have been identified, reflecting the growing body of research in the region. Several surveys reported 27 and 20 species in the central and western sections of northern Algeria, respectively [49,50]. In the south, mosquitoes had the attention of researchers due to the emergence of many malaria outbreaks [51,52]. In the northeast of the country, however, it has only gained research interest in the last few decades [53]. Studies on mosquitoes in various regions in Algeria have increased the total number of known species in the country, expanding our knowledge on the regional distribution of different species of *Culex*, *Culiseta*, and *Aedes* [54,55,56,57,58,59,60,61,62,63,64]. More recent work by Arroussi et al. [61] in the Annaba district identified eight species from four genera, including *Cx. pipiens*, *Ae. albopictus*, *Ae. aegypti*, *An. labranchiae*, and *Cs. longiareolata*. The present study expanded the geographic distribution of several species in the region and highlighted important spatial and temporal variations in their abundance.

*Aedes albopictus* has been reported in various regions in Algeria during the last two decades, suggesting a rapid range expansion. Its presence has been associated with declines in abundance of resident mosquito species like *Culex pipiens* and the absence of *Culiseta longiareolata*, which according to Carrieri, et al. [65] are prone to interspecific competition for resources. Brunhes, et al. [66] suspected that *A. albopictus* had arrived in Algeria after being established in Italy [67] and Albania [68]. Its introduction into Algeria was later confirmed by Izri, et al. [69] and Lafri, et al. [55]. Later, the species continued to spread in all Algeria [38,64,70], and rapidly became the second most abundant species after *Culex pipiens*. *Aedes aegypti* was widely distributed in Algeria along the coast [71] and was found as far south as Ghardaia [72]. The last recorded sighting of this species occurred in 1961 [73]. The low abundance of this species in our samples confirms the low frequency of the species in the region. The sporadic distribution of *Aedes aegypti* seems to have no link to climate change nor to the use of pesticides to combat malaria, but it could be related to a marked improvement in urban hygiene [74]. This species had not been recorded before 2021 in western Annaba, where it occurred at low abundance [61]. We found this species mostly at the end of summer and during early autumn, as was reported by Senevet and Andarelli [75].

The variation in mosquito species diversity observed across different sites at Fetzara Lake can be attributed to several environmental factors, notably the influence of land use types and the resulting differences in the physicochemical composition of water [6]. Sites near urban areas (e.g., NW and NE) tend to have altered water quality, often characterized by higher levels of pollutants due to runoff from roads, infrastructure, and industries [15,34]. These sites attracted both *Aedes geniculatus* and *Anopheles labranchiae*. On the other hand, sites near agricultural lands (e.g., SW and SE) are typically influenced by the use of fertilizers, pesticides, and irrigation practices, which can enrich the site with organic and mineral nutrients like nitrogen and phosphorus [76,77], which can create more favorable conditions for certain mosquito species such as *Culex pipiens*, *Culex perexiguus*, and *Aedes aegypti*. *Cs. longiareolata*, which is an ornithophilous mosquito [78], was found in the areas where there is poultry farming (NW and SE), suggesting that land use shapes species distribution.

We found seasonal variations in mosquito assemblages across sites, revealing that species composition and abundance vary considerably in space and time (season) within the same wetland. Studies have shown similar seasonal variability in mosquito assemblages of Mediterranean wetlands [79,80]. Similarly to studies in the tropical region [81], species richness increased during the dry season (summer). Although the phenology of most species aligned with previously documented patterns, we found that *Cs. longiareolata* was monophasic, appearing in the spring season (March–April–May), which is different than the polyphasic reproductive pattern (distributed throughout the year) reported by Merabti, et al. [82] in the Biskra region (Southeast Algeria). Such a difference is likely due to the major difference in climatic condition between the two regions. Our study adds new insights into the existing literature on seasonal variations in species composition and abundance in Mediterranean wetlands, underscoring the importance of fine-scale sampling of mosquito assemblages.

The stability in Culicidae diversity across years suggests that mosquito community composition remained relatively unchanged, likely due to consistent climatic conditions in the region. Temperature and precipitation are two key environmental factors influencing mosquito diversity, as they directly affect breeding, survival, and dispersal patterns [83]. In Central Sweden’s lower River Dalälven region, a study across different wetland types over three years revealed that species richness of mosquito remained relatively constant, albeit with variability in abundance between years [84]. Temperature plays a crucial role in mosquito life cycles, influencing larval development rates, adult longevity, and reproductive success [79,85]. Warmer temperatures generally accelerate development, leading to faster population turnover, while extreme heat can be detrimental to survival [86,87]. Precipitation determines the availability of aquatic habitats for mosquito breeding [88]. Increased rainfall can create temporary water bodies favorable for certain species, while prolonged dry periods may reduce breeding sites and limit population growth [89]. However, stable precipitation patterns across years suggest a consistent availability of suitable habitats, further supporting a stable mosquito community composition.

Despite our findings, the study has some limitations. Because we used ovitraps to sample mosquitoes, the collected assemblage can overlook species that develop in other types of substrates. Notably, genera such as *Anopheles* and some *Aedes* species prefer ground pools, marshes, or other natural water bodies, which could thus avoid egg laying in ovitraps. As a result, the findings presented here could represent only a subset of the broader mosquito community and may underestimate overall diversity. Nevertheless, our standardized approach can still document potential spatiotemporal variation in mosquito assemblages in the wetland.

This two-year study provides an extensive assessment of mosquito assemblage diversity and composition in the Ramsar site Fetzara Lake, highlighting the potential role of local habitat variability and seasons in shaping Culicidae assemblages. Our findings confirm that mosquito diversity and composition vary significantly across different parts of the wetland, likely due to differences in environmental conditions and land use. However, no differences were observed between the two studied years, suggesting a stable community structure driven by consistent climatic conditions. This study underscores the importance of considering spatial variability in mosquito research, particularly in complex wetland ecosystems. Future research should explore finer-scale environmental factors, such as water quality parameters and land use practices, to better understand their influence on mosquito communities. Also, long-term monitoring will be crucial to detecting potential shifts in species composition and vector-borne diseases under changing climatic and ecological conditions.

## Figures and Tables

**Table 1:** Average, SD, minimum, maximum and sample size (N) of the abundance of mosquito larvae collected in Fetzara lake.

Species	Code	Region	Site	Mean	SD	Min	Max	N
*Aedes aegypti*	AeEg	SW	S4	0.02	0.19	0	2	143
*Aedes albopictus*	AeAl	NE	S2	29.90	70.00	0	606	143
*Aedes albopictus*	AeAl	NW	S1	2.97	7.64	0	49	143
*Aedes albopictus*	AeAl	SE	S3	2.15	13.70	0	152	143
*Aedes albopictus*	AeAl	SW	S4	7.25	21.40	0	219	143
*Aedes geniculatus*	AeGn	NE	S2	0.15	1.40	0	16	143
*Aedes geniculatus*	AeGn	NW	S1	0.62	4.66	0	49	143
*Anopheles labranchiae*	AnLa	NE	S2	1.49	7.20	0	58	143
*Anopheles labranchiae*	AnLa	NW	S1	0.69	4.95	0	52	143
*Anopheles labranchiae*	AnLa	SE	S3	0.15	1.20	0	13	143
*Anopheles labranchiae*	AnLa	SW	S4	0.07	0.41	0	3	143
*Culex perexiguus*	CxPx	NE	S2	2.51	18.60	0	183	143
*Culex perexiguus*	CxPx	NW	S1	4.61	28.70	0	298	143
*Culex perexiguus*	CxPx	SE	S3	19.90	120.00	0	1252	143
*Culex perexiguus*	CxPx	SW	S4	3.20	22.30	0	236	143
*Culex pipiens*	CxPp	NE	S2	0.14	1.59	0	19	143
*Culex pipiens*	CxPp	NW	S1	26.90	67.00	0	455	143
*Culex pipiens*	CxPp	SE	S3	150.00	252.00	0	1604	143
*Culex pipiens*	CxPp	SW	S4	51.40	138.00	0	948	143
*Culiseta longiareolata*	CtLg	NW	S1	2.92	11.40	0	76	143
*Culiseta longiareolata*	CtLg	SE	S3	0.36	2.86	0	32	143

NE: Northeast; NW: Northwest; SE: Southeast; and SW: Southwest.

**Figure A1:**
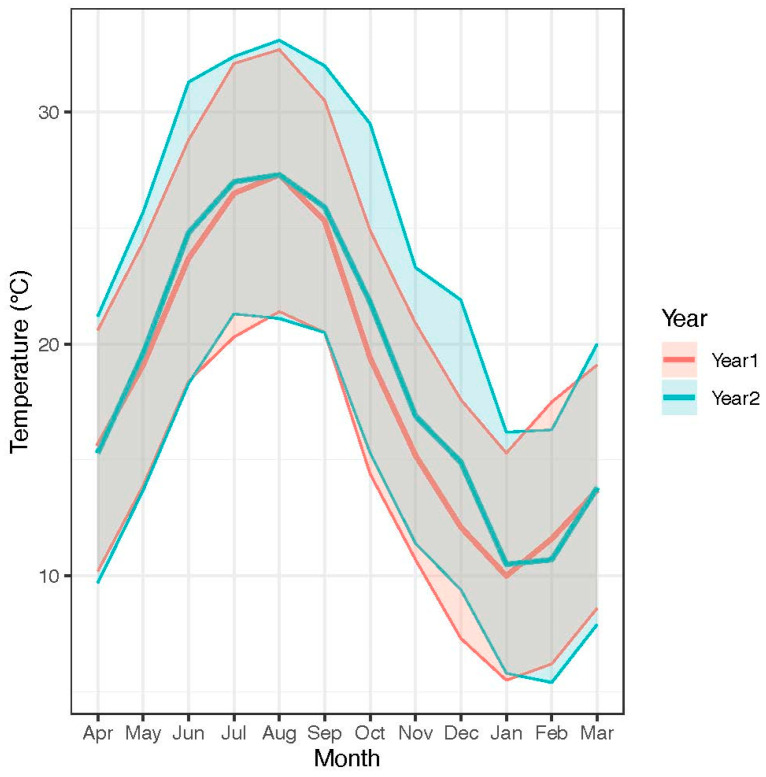
Seasonal pattern of air temperature in the region of Annaba in year 1 (2021–2022) and year 2 (2022–2023) of sampling. Values are monthly average temperature (bold line), corresponding to the average of daily mean temperature. The extremes (thin line) are minimum and maximum temperature.

## Data Availability

Data supporting this study are available from the authors upon reasonable request (rouibiam23@gmail.com).

## Appendix A

Weather. Average temperature during the studied period was 18.3 ± 6.2 °C in year 1 (2021–2022) and 19.0 ± 6.2 °C in year 2 (2022–2023) (Figure A1). There was no significant difference in the monthly temperature between the two years (W = 65.5, *p* = 0.73). Monthly precipitation averaged of 42.9 ± 36.0 mm in 2021–2022 and 28.6 ± 33.4 mm in 2022–2023. Monthly precipitation was not significantly different between the two years (W = 93.5, *p* = 0.225).
